# Body Fat Plays an Important Role in of Bioimpedance Spectroscopy-Based Dry Weight Measurement Error for Patients with Hemodialysis

**DOI:** 10.3390/diagnostics11101907

**Published:** 2021-10-15

**Authors:** Hae-Ri Kim, Jae-Wan Jeon, Hong-Jin Bae, Jin-Ah Shin, Young-Rok Ham, Ki-Ryang Na, Kang-Wook Lee, Dae-Eun Choi, Yun-Kyong Hyon

**Affiliations:** 1 Department of Nephrology, Chungnam National University Sejong Hospital, Sejong 30099, Korea; yo0118@cnuh.co.kr (H.-R.K.); jeonjwan@cnuh.co.kr (J.-W.J.); 2 Department of Nephrology, Cheongju St. Mary’s Hospital, Cheongju 28323, Korea; cnureddust@naver.com; 3 Department of Medical Science, Medical School, Chungnam National University, Daejeon 35015, Korea; wlsdkahh@gmail.com; 4 Nephrology, Medical School, Chungnam National University, Daejeon 35015, Korea; youngrok01@cnuh.co.kr (Y.-R.H.); drngr@cnu.ac.kr (K.-R.N.); kwlee@cnu.ac.kr (K.-W.L.); 5 Division of Industrial Mathematics, Data Analytics Team, National Institute for Mathematical Sciences, Daejeon 34047, Korea

**Keywords:** hemodialysis, dry weight, bioimpedance spectroscopy

## Abstract

Accurate dry weight (DW) estimation is important for hemodialysis patients. Although bioimpedance spectroscopy (BIS) is commonly used to measure DW, the BIS-based DW frequently differs from the clinical DW. We analyzed the characteristics of patients whose BIS-based DWs were over- and underestimated. In this retrospective cohort study, we evaluated 1555 patients undergoing maintenance hemodialysis in Chungnam National University Hospital. The gap (DWCP-BIS) was calculated by comparing the BIS and clinical DWs. We analyzed the clinical characteristics of patients with positive (*n* = 835) and negative (*n* = 720) gaps. Compared with other patients, the DWCP-BIS-positive group had higher extracellular water (ECW) level and extracellular/intracellular water index (E/I) and had lower weight, body mass index (BMI), lean tissue index (LTI), fat tissue index (FTI), fat mass (FAT), and adipose tissue mass (ATM). The DWCP-BIS-negative group exhibited elevated BMI, FTI, FAT, and ATM; however, it had lower height, ECW, and E/I. Linear regression analysis revealed that FAT significantly predicted DWCP accuracy. The clinical DW of patients with a low fat mass tended to be underestimated, while the clinical DW of patients with comparatively large fat reserves tended to be overestimated. These characteristics will aid in the reduction of BIS-based DW errors.

## 1. Introduction

Appropriate volume management is important in patients undergoing hemodialysis. It can be difficult to accurately determine the DW, which comprises the lowest acceptable post-dialysis weight associated with minimal signs or symptoms of hypovolemia or hypervolemia [1]. In hemodialysis patients, overhydration (OH) and dehydration trigger many adverse events. OH is associated with congestive heart failure, hypertension, increased arterial stiffness, left ventricular hypertrophy, cardiovascular events, and increased mortality and morbidity [2,3,4]. Dehydration causes cramping, vomiting, nausea, dizziness, intradialytic hypotension, post-dialysis orthostatic hypotension, organ damage, and functional impairments (e.g., ischemic heart and brain disease) [5,6].

Several methods are used to measure DW, including measurements of blood and jugular venous pressure, as well as edema status. However, these methods do not consider underlying illnesses or any reduction in muscle mass [7]. DW has also been assessed by referring to atrial or brain natriuretic peptide levels, ultrasound-measured inferior vena cava diameter, and blood pressure measurements, lung ultrasound [8]. However, these DWs are inaccurate [9,10,11]. Recently, multifrequency bioimpedance analysis, also termed bioimpedance spectroscopy (BIS), has been used to determine body water levels [12]. BIS yields information regarding fat, lean, and cell mass distributions, as well as intracellular, extracellular, and interstitial water volumes. BIS assesses the volume status of hemodialysis patients and measures DW [12,13]. Although DW data are fairly accurate, BIS-based and clinical DWs often differ. Approximately 24–27% of dialysis patients are reportedly overhydrated, despite BIS-based monitoring of water levels [14,15]. BIS-assessed fluid overload increases mortality and hypertension [16]. In particular, malnutrition-related factors have been associated with OH in hemodialysis patients. Here, we analyzed the characteristics of patients for whom BIS over- or underestimated DW; we also sought to accurately predict the clinical DW.2. 

## 2. Materials and Methods

### 2.1. Study Design

This was a single-center retrospective study. All data were retrieved from the medical records of Chungnam National University Hospital. Mortality data were those of the National Health Insurance Service database (IRB approval no. 2021-01-007).

### 2.2. Study Population

All screened patients had end-stage renal disease and underwent outpatient hemodialysis from January 2016 to June 2020. Patients who underwent hemodialysis at least three times weekly were enrolled. All were adults over 18 years of age. Patients were excluded if they had unstable clinical conditions (i.e., acute infections), if they were missing hydration data or BIS DW data, if their clinical DWs could not be confirmed, if they underwent dialysis less than twice weekly, if they had gaps between BIS and clinical DWs of <–10 kg or >10 kg, and if their BIS data were obtained immediately after hemodialysis.

### 2.3. Body Composition

Body composition including hydration status (i.e., OH) was assessed using a portable BIS device (BCM; Fresenius Medical Care, Bad Homburg, Germany). Data were collected from pre-dialysis times performed on the second or third session dialysis day of the week. The BIS data included the extracellular water (ECW), intracellular water (ICW), and total body water (TBW) levels; the LTI, FTI, ECW/ICW ratio (E/I), lean tissue mass (LTM), fat mass (FAT), adipose tissue mass (ATM), and body cell mass (BCM). The ECW, ICW, and TBW were calculated using a fluidic model 20. These fluid volumes were then used to determine fluid overload, expressed as OH values. The OH was either negative or positive. ECW, ICW, TBW, and OH were all expressed in liters.

### 2.4. Definitions

A previous study defined the DW as the lowest weight after dialysis associated with minimal symptoms or signs of dehydration or OH. We used the concept of clinically appropriate DW (DWCP), which differed from the BCM DW (DWBIS). The DWCP was the post-dialysis weight associated with no sign or symptom of OH or dehydration, during or after dialysis. The DW was determined via clinical evaluation by referring to the BIS-predicted DW. Clinical judgments were made by referring to patient parameters and imaging data. For example, OH was characterized by peripheral and generalized edema, chest discomfort, or pleural effusion/pulmonary edema not evident on a chest X-ray. Dehydration was associated with hypotension onset during or after dialysis. We also evaluated muscle spasm and dizziness status. In patients who developed hypotension during dialysis, patients who used intravenous fluid prophylactically were also included as occurrences of dehydration The BIS OH value was termed the OHBIS. The BIS-assessed DW (DWBIS) was the body weight minus the OHBIS value (kg). The gap between the DWBIS and the clinical DW (DWCP) was the dry weight difference (DWCP-BIS) and thus the body weight minus the difference in the DWBIS and DWCP (kg). Each DWCP-BIS was either positive or negative. If positive, the DWCP was higher than the DWBIS, and the DWBIS was presumed to underestimate the DW. When the DWCP-BIS was negative, the DWBIS was presumed to overestimate the DW.

### 2.5. Assessment of DWCP

Basically, the medical staffs of the hemodialysis center where research has been co ducted check all symptoms, signs, and events that have occurred since the previous dialysis before the visit when the patient visits the hospital on the day of hemodialysis and writes them down in the electronic medical record. The stability of all vital signs measured before dialysis is checked, and abnormal symptoms or signs are checked through physical examination, palpation, auscultation, and history taking. In addition, blood pressure during dialysis is basically measured every hour, but if dry weight decreases or increases through body composition measurement, blood pressure is measured every 30 min during dialysis. When vital signs become unstable, when blood pressure fluctuations are severe, or when a patient complains of symptoms of hypotension, this information is systematized to notify the attending physician immediately. When setting a new dry weight, patient’s condition is checked for at least 2 weeks and 200–300 g changed for each dialysis session; clinical signs judged by the medical staff and subjective symptoms of which the patient complained were combined to set the weight after dialysis to keep the patient in a stable condition. In addition, after changing the dry weight, the presence of pulmonary edema or pleural effusion was checked through a chest X-ray. Continuous chest X-rays were performed on every dialysis day when excessive body water was suspected depending on the patient. The stable DWCP was determined after assessing patient stability during and between dialysis using new dry weights for at least 2 weeks.

### 2.6. Clinical Parameters

We recorded age at commencement of dialysis (in days), sex, height (cm), initial body weight (kg), initial systolic and diastolic blood pressures (mmHg), and any comorbidity (diabetes mellitus and/or hypertension). All laboratory tests were performed prior to dialysis, within 3 days before or after DWBIS measurements. The following levels were measured: hemoglobin, blood urea nitrogen, serum creatinine, serum albumin, serum total calcium, serum phosphorus, serum sodium, serum potassium, and serum chloride.

### 2.7. Outcomes

We divided patients into two groups according to the presence of positive or negative DWCP-BIS values. Both groups were then divided into three subgroups on the basis of the DWCP-BIS: less than 1 kg, more than 1 kg but less than 2 kg, and more than 2 kg; and less than 0 but more than –1 kg, less than –1 kg but more than –2 kg, and less than 2 kg. We sought clinical and BCM parameters that affected the DWCP-BIS values. Correlations between parameters or features were derived, and the magnitudes of effects were predicted by referring to the correlations.

### 2.8. Statistical Analyses

All data are presented as means with standard deviations; a *p*-value < 0.05 was considered to indicate statistical significance. *p*-values were obtained using one-tailed and paired t-tests. Box plots were drawn to reveal the interquartile trends of various parameters/features of the three groups. We performed linear regression analyses of fat, muscle and other parameters; we explored whether any parameter predicted the DWCP. Predictive accuracies were evaluated as the mean of 20 predictions; all test sets were subsamples of the full dataset.

## 3. Results

### 3.1. Baseline Characteristics

**Figure 1:**
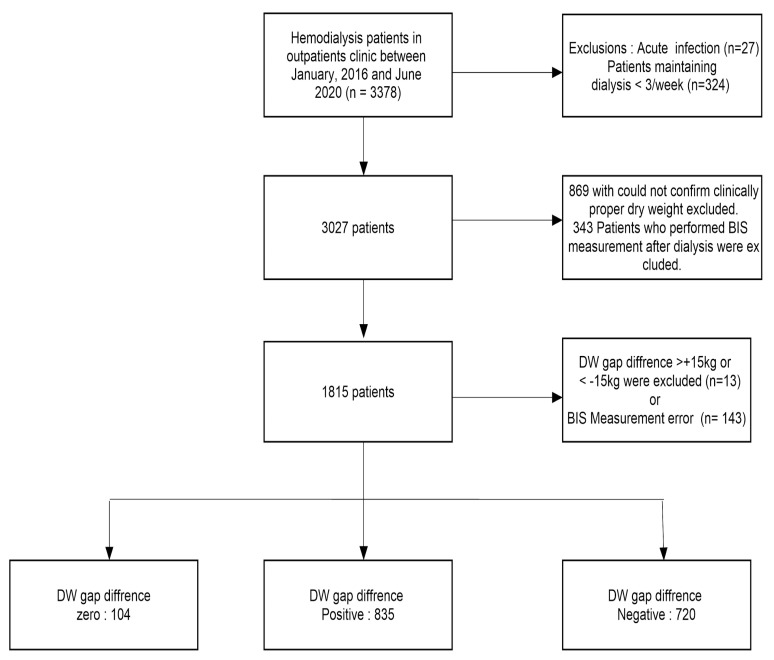
Study population.

In total, 3378 patients underwent outpatient dialysis in Chungnam National University Hospital from January 2016 to June 2020. The following patients were excluded: patients with acute infections at the time of BIS evaluation (*n* = 27), patients whose DWCP values were not recorded after BIS (*n* = 869), patients who underwent dialysis less than twice weekly (*n* = 324), patients who underwent BIS after dialysis (*n* = 343), patients with DWCP-BIS values > 15 kg and <–15 kg (*n* = 13), patients whose BIS parameters were lacking or extreme (*n* = 143), and patients with DWCP-BIS values of zero (*n* = 95). Finally, 1555 patients were analyzed, of whom 835 were in the DWCP-BIS-positive group and 720 were in the DWCP-BIS-negative group. The mean ages were 64–65 years in both groups; patients in the DWCP-BIS-positive group were shorter and weighed less, compared with patients in the DWCP-BIS-negative group. The positive DWCP-BIS group underwent 835 tests (equal to the number of patients) and the negative DWCP-BIS group underwent 720 tests (also equal to the number of patients; Figure 1). Compared with all patients, patients in the DWCP-BIS-positive group exhibited greater height, ECW, and E/I values; however, they exhibited lower weight, hemoglobin, total protein, albumin, phosphorous, BMI, LTI, FTI, FAT, and ATM values. Compared with all patients, patients in the DWCP-BIS-negative group exhibited higher hemoglobin, total protein, albumin, phosphorous, BMI, FTI, FAT, and ATM values; however, they exhibited lower height, ECW, and E/I values (Table 1).

### 3.2. Clinical Differences between the Two Groups

We compared clinical features between the DWCP-BIS-positive and -negative groups. Patients in the DWCP-BIS-positive group were significantly taller, weighed less, and had lower BMI, FAT, FTI, and a lower albumin level, as well as higher E/I values, compared with patients in the DWCP-BIS-negative group in both absolute DWCP-BIS values < 1 kg patients and absolute DWCP-BIS values > 2 kg patients. However, patients in the DWCP-BIS-positive group exhibited lower hemoglobin, total protein, blood urea nitrogen, creatinine, and phosphorous levels; lower LTI and ICW values; and a higher ECW value, compared with patients in the DWCP-BIS-negative group in absolute DWCP-BIS values > 2 kg patients (Table 2, Supplementary Table S1 and Figure S1).

### 3.3. Clinical Characteristics as DWBIS and DWCP Varied

**Figure 2:**
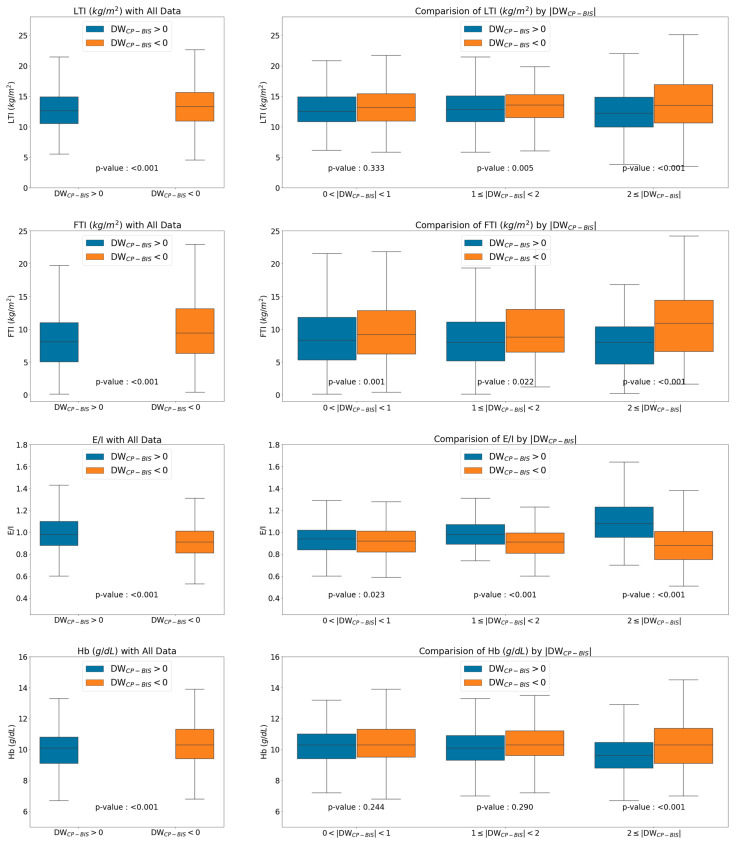

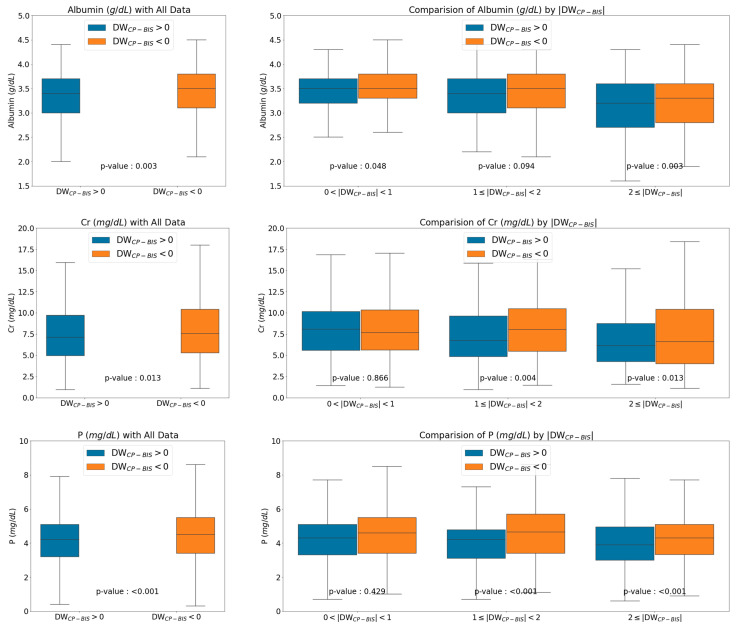
Comparison of clinical parameter by differences of DW_CP-BIS_.

We analyzed the clinical characteristics according to the difference between the DWBIS and DWCP, thus ≤1 kg and ≥1–2 kg (Table 2). Among patients in the DWCP-BIS-negative group, those with values < 1 kg (compared with those who had values > 2 kg) were older and had higher body weight and higher BMI. When the |DWCP-BIS| was >2 kg, the muscle and fat masses tended to be higher. Although both ECW and ICW were elevated, the increase in the ICW was larger and the E/I was low; the albumin level was also significantly lower. Among patients in the DWCP-BIS-positive group, those with values ≥ 2 kg (compared with those who had values < 1 kg) were younger, and their weight and BMI both tended to be lower. Among patients with DWCP-BIS values > 2 kg, muscle and fat mass-related factors tended to be lower, and the ICW was lower; in contrast, the ECW and E/I were high. The hemoglobin, total protein, albumin, blood urea nitrogen, creatinine, phosphorous, and potassium levels were significantly reduced among patients in the group with DWCP-BIS values > 2 kg (Table 2, Figure 2, Supplementary Table S2 and Figure S2). 

### 3.4. Prediction of Clinical DW in Patients with Positive and Negative DWCP-BIS Values

We used linear regression to explore the effects of muscle-related factors (LTI [kg/m^2^] and LTM [kg]) and fat-related factors (ATM [kg]), FAT [kg], and FTI [kg/m^2^]) on predictive accuracy. The prediction was performed with 20 random samples, independently (Table 3). For each sample, the train and test set are exclusively chosen with the ratio 8:2. The accuracy and mean absolute error for each prediction are obtained by taking mean value of the 20 experiments with the samples. DWCP was better predicted among patients in the DWCP-BIS-negative group than among patients in the DWCP-BIS-positive group. Regression analysis excluding ATM, FAT, and FTI reduced the accuracy of DWCP prediction, compared with analyses that included all factors (for both DWCP-BIS-negative and -positive groups) (Table 4). Regression analysis excluding the LTI and LTM predicted the DWCP with accuracy similar to that of analyses that included all factors (for both DWCP-BIS-negative and -positive groups), as did analyses including only BIS factors (Table 5).

## 4. Discussion

In this study, we showed that body fat mass is related to the DWCP prediction error based on BIS. Generally, DW is defined as the lowest weight measured after dialysis that is associated with minimal symptoms or signs of dehydration or overhydration [1,17,18]. Thus, DW determination methods are subjective, inconsistent, and non-reproducible. Recently, BIS has shown non-invasive, yielding comparatively consistent DWs by measuring ICW and ECW levels [19,20]. However, it has been reported that the BIS based DW prediction does not match the DWCW in some hemodialysis patients. An overestimated DWBIS (associated with a negative DWCP-BIS) is higher than the DWCP; in this situation, edema, chest discomfort, dyspnea, and uncontrolled hypertension may develop [21,22,23]. An underestimated DWBIS (associated with a positive DWCP-BIS) is lower than the DWCP; in this situation, hypotension, cramping, dizziness, and altered consciousness may develop [24,25]. In our study, patients in our positive DWCP-BIS group exhibited malnutrition, and were taller and thinner, with a lower BMI and fat mass, compared with patients in the negative DWCP-BIS group. The lower E/I and higher fat mass created a large gap in the negative DWCP-BIS group. The higher E/I and lower fat mass created a large gap in the positive DWCP-BIS group. E/I was negatively associated with both BMI and adipose tissue mass. The muscle mass did not significantly affect the gap. Linear regression revealed that fat mass significantly predicted the accuracy of the DWCP, thus predicting the DWCP-BIS.

The exact mechanism by which BIS showed error in predicting clinical DW in patients with relatively high or low fat mass is unknown. However, two factors can be considered. First, high fat mass may enhance cellular resistance, reducing the accuracy of BIS measurements [26]. Second, BIS estimates the volume status based on data obtained from healthy individuals. Therefore, patients with a BMI that is too high or a low BMI or who are undergoing hemodialysis may have different results. Carter et al. reported that BIS volume prediction was poor in patients with very high or very low BMI [27]. Low fat content is associated with high levels of TBW [28]. Obese individuals typically present with edema and relatively high TBW and E/I value [26,29]. In hemodialysis patients, ECW volume is negatively associated with BMI [30].

In our study, the positive DWCP-BIS group showed lower hemoglobin, total protein, blood urea nitrogen, creatinine, and phosphorous levels and lower LTI compared to the negative DWCP-BIS group in 2 kg < |DWCP-BIS| not 1 kg > |DWCP-BIS| patients. These indicate malnutrition leads to larger dry weight errors in positive DWCP-BIS group. However, as |DWCP-BIS| increases, there can be seen possibility of imbalances between negative and positive groups in Table 2. If there is no limitation of number of data, more rigorous conclusions can be obtained, especially on |DWCP-BIS| > 2 kg. In patients with malnutrition, protein levels are low, which is associated with reduced intravascular oncotic pressure and edema involving the extracellular interstitium [31]. Muscles are important reservoirs of water and play important roles in water circulation [32]. Thus, a reduction in muscle volume will change the extracellular fluid level. 

In our study, albumin levels were low in both DWCP-BIS-negative and -positive groups. Compared with patients in the DWCP-BIS-negative group, the extent of DWCP-BIS-positivity increased as the albumin level rose. The total protein (albumin) level greatly affected blood osmotic pressure, and was therefore a critical clinical factor in both groups. Hypoalbuminemia is associated with an increase in the ECW level and a decrease in the ICW level [33,34,35]. Although we found that hypoalbuminemia lacked clinical significance, the DWCP-BIS values in both groups increased as hypoalbuminemia became more pronounced. Thus, in such patients, the DWBIS will differ from the DWCP.

This study has several limitations. First, although the statistical tendencies of all patients were analyzed, our study cannot completely explain the effect association between the definite cause of the dry weight gap of patients and the parameters because of retrospective observational study. DWCP is individualized for each patient, and some patients did not match the common trend of this study. In these patients, DWCP may not be a perfect value. Second, parameters related to inflammation were not collected and the effect of inflammation on the dry weight gap was not analyzed. Third, intradialytic hypotension (an inappropriate measure of DW) was not reliably assessed because the use of blood pressure medication before dialysis was not controlled. Forth, data regarding patients with ascites and congestive heart failure (caused by liver failure) may be unreliable because stable body weights were recorded prior to edema development. Fifth, because the measurement of BIS was not performed regularly, longitudinal analysis was not performed. In the future, if periodic BIS measurement is carried out through a prospective study, more accurate data can be collected. Moreover, we did not examine data from peritoneal dialysis patients; further investigations are required involving these patients.

In conclusion, BIS-based DWs may be over- or underestimated, due to the influences of fat content and serum albumin. DWBIS values may underestimate the clinical DW in patients with low fat mass and malnutrition and overestimate the clinical DW in patients with high fat mass and BMI. In the future, research to establish BIS reference in people with abnormal BMI and fat mass will be needed. 

## Figures and Tables

**Table 1:** Baseline characteristics between positive and negative of DW_CP-BIS_.

Group	Positive (*n* = 835)		Negative (*n* = 720)		
Feature	Mean	SD	Mean	SD	*p*-Value *
Age	65.4	11.85	64.66	12.69	0.204
Gender	302 (36%)	0.48	329 (46%)	0.5	0.002
Height (cm)	162.56	8.59	160.03	8.22	<0.001
Weight (kg)	59.44	10.74	61.74	11.88	<0.001
Vintage of HD (day)	839.15	850.65	803.61	799.5	0.007
DM	527 (63%)	0.48	408 (57%)	0.5	0.027
HTN	672 (80%)	0.4	672 (79%)	0.41	0.227
Hb (g/dL)	9.99	1.52	10.31	1.54	<0.001
Total Protein (g/dL)	6.28	0.73	6.41	0.66	0.006
Albumin (g/dL)	3.33	0.57	3.44	0.59	0.003
BUN (mg/dL)	54.75	23.45	56.9	23.07	0.02
Cr (mg/dL)	7.52	3.24	7.92	3.54	0.013
Tca (mg/dL)	8.36	0.8	8.36	0.77	0.345
P (mg/dL)	4.21	1.55	4.49	1.58	<0.001
Na (mEq/L)	136.95	3.98	137.14	3.96	0.431
K (mEq/L)	4.74	0.95	4.73	0.89	0.559
Cl (mEq/L)	101.09	5.07	101.46	5.23	0.143
TBW (L)	32.19	7.07	31.79	7.23	0.521
ECW (L)	15.98	3.54	15.03	3.44	<0.001
ICW (L)	16.2	4.07	16.74	4.37	0.002
E/I	1.01	0.19	0.92	0.18	<0.001
BMI (kg/m^2^)	22.43	3.53	24.05	3.97	<0.001
LTI (kg/m^2^)	12.77	3.31	13.38	3.56	<0.001
FTI (kg/m^2^)	8.35	4.33	10	4.86	<0.001
LTM (kg/m^2^)	34.11	10.54	34.69	11.1	0.108
FAT (kg)	17.31	9.22	20.17	9.96	<0.001
ATM (kg)	20.44	10.42	23.59	11.2	<0.001
BCM (kg)	18.64	7.13	19.27	7.52	0.021
DW_BIS_ (kg)	56.55	10.48	60.54	11.68	<0.001
OH(L)_BIS_ (kg)	2.84	2.38	1.27	2.14	<0.001
DWCP (kg)	58.29	10.32	59.14	11.56	0.123
DW_CP-BIS_ (kg)	1.75	2.05	−1.4	1.9	<0.001
OH(L)_CP_ (kg)	1.15	2.63	2.6	2.59	<0.001

* *p*-value positive vs. negative. Abbreviations: SD, standard deviation; HD, Hemodialysis; DM, diabetes mellitus; HTN, hypertension; Hb, hemoglobin; BUN, blood urea nitrogen; Cr, serum creatinine; TCa, serum total calcium; P, serum phosphorus; Na, sodium; K, serum potassium; Cl, serum chloride; TBW, total body water; ECW, extracellular water; ICW, intracellular water; E/I, extracellular water to intracellular water ratio; BMI, body mass index; LTI, lean tissue index; FTI, fat tissue index; lean tissue mass, LTM; FAT, fat mass; ATM, adipose tissue mass; BCM, body cell mass; DW, dry weight; OH, overhydration.

**Table 2:** Clinical characteristics as subdivision of DW_CP-BIS_.

Group	0 kg < |DW_CP-BIS_| < 1 kg					1 kg ≤|DW_CP-BIS_|< 2 kg					2 kg ≤ |DW_CP-BIS_|				
	Negative (*n* = 434)		Positive (*n* = 405)			Negative (*n* = 132)		Positive (*n* = 184)			Negative (*n* = 154)		Positive (*n* = 246)		
Feature	Mean	SD	Mean	SD	*p*-Value	Mean	SD	Mean	SD	*p*-Value	Mean	SD	Mean	SD	*p*-Value
Age	63.76	12.66	65.89	12.18	0.005	65.63	12.00	66.59	11.23	0.308	66.34	13.17	63.72	11.64	0.204
Gender (%)	204 (47%)	0.50	157 (39%)	0.49	0.005	60 (45%)	0.50	65 (35%)	0.48	0.469	65 (42%)	0.50	80 (33%)	0.47	0.002
Height (cm)	159.88	8.40	161.94	8.03	<0.001	159.41	7.51	162.14	8.65	<0.001	160.96	8.25	163.88	9.29	0.016
Weight (kg)	60.78	11.47	59.61	11.23	0.232	61.48	11.29	59.55	9.70	0.053	64.68	13.08	59.07	10.68	<0.001
Vintage of HD (day)	745.55	753.38	874.61	848.06	0.095	934.81	868.91	769.04	795.23	0.095	854.78	850.92	833.20	893.90	0.938
DM	239 (55%)	0.50	247 (61%)	0.49	0.066	74 (56%)	0.50	119 (65%)	0.48	0.066	95 (62%)	0.49	161 (65%)	0.48	1
HTN (%)	351 (81%)	0.39	343 (85%)	0.36	0.178	104 (79%)	0.41	148 (80%)	0.40	0.178	111 (72%)	0.45	181 (74%)	0.44	0.607
Hb (g/dL)	10.33	1.44	10.21	1.39	0.244	10.25	1.74	10.10	1.51	0.290	10.29	1.63	9.54	1.64	<0.001
Total Protein (g/dL)	6.41	0.66	6.35	0.66	0.188	6.48	0.57	6.34	0.65	0.068	6.35	0.72	6.11	0.86	0.006
Albumin (g/dL)	3.51	0.56	3.44	0.54	0.048	3.40	0.50	3.35	0.47	0.094	3.26	0.68	3.13	0.63	0.003
BUN (mg/dL)	57.68	22.61	57.86	23.17	0.654	55.25	22.02	51.54	22.83	0.033	56.10	25.18	52.02	23.79	0.020
Cr (mg/dL)	8.07	3.35	8.08	3.17	0.866	8.04	3.40	7.29	3.17	0.004	7.40	4.08	6.76	3.26	0.020
Tca (mg/dL)	8.36	0.76	8.38	0.74	0.985	8.30	0.82	8.32	0.71	0.579	8.40	0.76	8.36	0.95	0.345
P (mg/dL)	4.49	1.49	4.38	1.61	0.429	4.71	1.76	4.08	1.49	0.001	4.32	1.63	4.03	1.47	0.001
Na (mEq/L)	137.19	3.92	137.10	4.04	0.913	136.90	3.95	136.71	3.62	0.308	137.19	4.12	136.89	4.13	0.431
K (mEq/L)	4.73	0.87	4.82	0.89	0.135	4.75	0.86	4.82	1.02	0.920	4.69	0.97	4.57	0.96	0.559
Cl (mEq/L)	101.66	5.29	101.27	5.08	0.470	100.48	5.05	101.13	5.16	0.761	101.73	5.14	100.78	5.00	0.143
TBW (L)	31.51	7.13	31.64	6.81	0.559	31.62	6.65	32.31	6.70	0.907	32.72	7.93	33.00	7.67	0.521
ECW (L)	14.99	3.48	15.24	3.20	0.166	14.97	3.00	16.05	3.29	0.028	15.16	3.69	17.14	3.92	<0.001
ICW (L)	16.47	4.10	16.38	4.05	0.968	16.65	4.18	16.24	3.82	0.030	17.57	5.15	15.88	4.30	<0.001
E/I	0.93	0.16	0.95	0.16	0.023	0.93	0.19	1.00	0.16	<0.001	0.90	0.22	1.11	0.21	<0.001
BMI (kg/m^2^)	23.73	3.75	22.62	3.85	<0.001	24.14	3.96	22.58	3.03	<0.001	24.87	4.45	21.99	3.28	<0.001
LTI (kg m^2^)	13.22	3.22	12.94	3.31	0.333	13.46	3.57	12.90	3.01	0.005	13.79	4.34	12.38	3.51	<0.001
FTI (kg/m^2^)	9.76	4.75	8.74	4.49	0.001	9.95	4.92	8.34	3.99	0.022	10.74	5.09	7.73	4.23	<0.001
LTM (kg/m^2^)	34.31	10.53	34.35	10.39	0.728	34.49	10.85	34.35	9.92	0.190	35.93	12.74	33.55	11.22	0.108
FAT (kg)	19.53	9.64	17.80	9.33	0.011	19.87	9.83	17.32	8.39	0.122	22.25	10.69	16.51	9.59	<0.001
ATM (kg)	22.86	10.53	21.47	10.94	0.052	23.58	11.55	20.05	9.29	0.020	25.65	12.47	19.04	10.22	<0.001
BCM (kg)	18.99	7.04	18.89	7.08	0.95	19.28	7.50	18.80	6.62	0.063	20.06	8.72	18.10	7.58	0.021
ECF/BMI (m^2^)	0.82	0.31	0.87	0.47	0.056	0.81	0.31	0.84	0.30	0.188	0.82	0.36	0.83	0.33	<0.001
ICW/BMI (L·m^2^/kg)	0.70	0.18	0.75	0.36	0.002	0.70	0.17	0.73	0.17	0.048	0.72	0.22	0.73	0.18	<0.001
ATM/BMI (m^2^)	0.94	0.35	0.95	0.68	0.397	0.95	0.37	0.87	0.35	0.654	1.01	0.43	0.86	0.42	0.023
LTM/BMI (m^2^)	1.48	0.47	1.58	0.79	<0.001	1.46	0.47	1.54	0.46	0.866	1.47	0.54	1.54	0.50	<0.001
FAT/BMI (m^2^)	0.80	0.32	0.78	0.52	0.038	0.80	0.33	0.75	0.32	0.985	0.88	0.36	0.74	0.38	<0.001
E/I/BMI (m^2^/kg)	0.04	0.01	0.04	0.02	0.005	0.04	0.01	0.05	0.01	0.429	0.04	0.01	0.05	0.01	0.105
DW_BIS_ (kg)	59.29	11.04	57.62	10.83	0.005	60.18	11.27	56.66	9.29	0.913	64.37	12.97	54.69	10.52	0.334
OH(L)_BIS_ (kg)	1.50	1.93	1.89	1.78	<0.001	1.26	1.76	2.84	1.98	0.135	0.63	2.78	4.38	2.70	0.016
DW_CP_ (kg)	58.90	11.04	58.04	10.84	0.232	58.78	11.27	58.11	9.31	0.47	60.13	13.15	58.85	10.17	<0.001
DW_CP-BIS_ (kg)	−0.40	0.25	0.41	0.26	0.095	−1.40	0.29	1.46	0.28	0.559	−4.24	2.39	4.16	2.30	0.938
OH(L)_CP_ (kg)	1.88	1.97	1.58	2.31	0.066	2.70	1.82	1.44	2.16	0.166	4.55	3.51	0.22	3.18	1

Abbreviations: SD, standard deviation; HD, Hemodialysis; DM, diabetes mellitus; HTN, hypertension; Hb, hemoglobin; BUN, blood urea nitrogen; Cr, serum creatinine; TCa, serum total calcium; P, serum phosphorus; Na, sodium; K, serum potassium; Cl, serum chloride; TBW, total body water; ECW, extracellular water; ICW, intracellular water; E/I, extracellular water to intracellular water ratio; BMI, body mass index; LTI, lean tissue index; FTI, fat tissue index; lean tissue mass, LTM; FAT, fat mass; ATM, adipose tissue mass; BCM, body cell mass; DW, dry weight; OH, overhydration.

**Table 3:** Features.

Feature Group (Number of Features)	Features
BIS (15)	Gender, Age, Height (cm), Weight (kg), TBW (L), ECW (L),ICW (L), E/I, BMI (kg/m^2^), LTI (kg/m^2^), FTI (kg/m^2^), LTM (kg), FAT (kg), ATM (kg), BCM (kg)
Non-BIS (Lab. and Nutrition) (12)	DM, HTN, Hb (g/dL), Total Protein (g/dL), Albumin (g/dL), BUN (mg/dL), Cr (mg/dL), Tca (mg/dL), P (mg/dL),Na (mEq/L), K (mEq/L), Cl (mEq/L)

**Table 4:** Comparison of prediction accuracy of DW_CP_ using linear regression by fat and muscle feature groups for −15 kg < DW_CP-BIS_ < 15 kg.

	Overestimation (Negative)		Underestimation (Positive)	
Selected Features (No. of Features)	Prediction Accuracy (%)	Mean Absolute Error (kg)	Prediction Accuracy (%)	Mean Absolute Error (kg)
All (27)	96.84	1.244	93.18	1.393
Excluding LTI (kg/m^2^), LTM (kg) (25)	97.12	1.223	93.35	1.383
Excluding ATM (kg), FAT (kg), FTI (kg/m^2^) (24)	94.73	1.258	91.77	1.442

**Table 5:** Comparison of prediction accuracy of DW_CP_ using linear regression by fat and muscle feature groups only on BIS features for −15 kg < DW_CP-BIS_ < 15 kg.

	Overestimation (Negative)		Underestimation (Positive)	
Selected Features (No. of Features)	Prediction Accuracy (%)	Mean Absolute Error (kg)	Prediction Accuracy (%)	Mean Absolute Error (kg)
BIS only (15)	96.94	1.243	92.92	1.406
Excluding LTI (kg/m^2^), LTM (kg) (13)	97.04	1.284	93.26	1.386
Excluding ATM (kg), FAT (kg), FTI (kg/m^2^) (12)	94.22	1.233	91.81	1.437

## Supplementary Materials

The following are available online at https://www.mdpi.com/article/10.3390/diagnostics11101907/s1. Table S1: Comparison between negative and positive groups in each of the same absolute degree of differences. Table S2: Comparison between less than 1 kg of GAP and more than 2 kg of GAP in negative and positive group, respectively. Figure S1: (A) Clinical Parameters as degrees of differences in Positive DW_CP-BIS_ groups. (B) Clinical Parameters as degrees of differences in Negative DW_CP-BIS_ groups. Figure S2: Comparison of clinical parameter by differences of DW_CP-BIS_.
