# Supplementary material for: Body Fat Plays an Important Role in of Bioimpedance Spectroscopy-Based Dry Weight Measurement Error for Patients with Hemodialysis
Source: Diagnostics (Basel). 2021 Oct 15;11(10):1907. doi: 10.3390/diagnostics11101907 (PMC8535067; doi:10.3390/diagnostics11101907)
Supplement: Supplementary file 1 [file diagnostics-11-01907-s001.zip › diagnostics-1388143-supplementary.pdf]

Table S1. Comparison between negatve and positive groups in each of the same absolute degree of differences.

	Pair T-test		
Group	0kg < DW_{CP-BIS} < 1kg Vs. -1kg < DW_{CP-BIS} < 0kg	1kg ≤ DW_{CP-BIS} < 2kg Vs. -2kg < DW_{CP-BIS} ≤ -0kg	2kg ≤ DW_{CP-BIS} Vs. -2kg ≤ DW_{CP-BIS}
Feature	p-value	p-value	p-value
Age	0.00539	0.308495	0.105076
Gender(%)	0.005076	0.468948	0.333599
Height (cm)	0.000101	0.016801	0.015952
Weight (kg)	0.231582	0.052842	2.30E-08
Vintage of HD (day)	0.095355	0.523764	0.938017
DM	0.065993	0.273175	1
HTN(%)	0.178249	0.367704	0.607181
Hb (g/dL)	0.244155	0.289652	0.000146
Total Protein (g/dL)	0.188359	0.067745	0.001999
Albumin (g/dL)	0.047522	0.094349	0.046939
BUN (mg/dL)	0.653583	0.032601	0.011525
Cr (mg/dL)	0.866093	0.004409	0.020969
Tca (mg/dL)	0.984873	0.579089	0.490851
P (mg/dL)	0.428641	1.46E-05	0.081678
Na (mEq/L)	0.912956	0.307781	0.899127
K (mEq/L)	0.135443	0.920169	0.113376
Cl (mEq/L)	0.469874	0.76082	0.292536
TBW (L)	0.558567	0.907086	0.633869
ECW (L)	0.166276	0.027937	0.000238
ICW (L)	0.968246	0.029826	0.000112
E/I	0.02324	1.14E-05	4.76E-14
BMI (kg/m^2)	5.20E-05	0.000289	1.45E-13
LTI (kg/m^2)	0.333201	0.004963	0.000786
FTI (kg/m^2)	0.001115	0.022047	1.03E-11
LTM (kg/m^2)	0.728308	0.189777	0.022232
FAT (kg)	0.011051	0.122354	2.55E-10
ATM (kg)	0.052484	0.019913	2.58E-10
BCM (kg)	0.950009	0.062997	0.010099
DW_{BIS} (kg)	0.056273	0.001144	1.41E-16
$OH(L)_{BIS}$ (kg)	0.001567	1.93E-10	2.28E-23
DW_{CP} (kg)	0.39706	0.341786	0.022542
DW_{CP-BIS} (kg)	1.99E-162	1.96E-112	2.18E-72
$OH(L)_{CP}$ (kg)	0.037836	9.10E-07	2.94E-25

Abbreviations: SD, standard deviation; HD, Hemodialysis; DM, diabetes mellitus; HTN, hypertension; Hb, hemoglobin; BUN, blood urea nitrogen; Cr, serum creatinine; TCa, serum total calcium; P, serum phosphorus; Na, sodium; K, serum potassium; Cl, serum chloride; TBW, total body water; ECW, extracellular water; ICW, intracellular water; E/I, extracellular water to intracellular water ratio; BMI, body mass index; LTI, lean tissue index; FTI, fat tissue index; lean tissue mass, LTM; FAT, fat mass; ATM, adipose tissue mass; BCM, body cell mass; DW, dry weight; OH, overhydration.

Table S2. Comparison between less than 1kg of GAP and more than 2kg of GAP in negative and positive group, respectively.

Group	Negative					Positive				
	-1kg < DW_{CP-BIS} <0kg (n=434)		DW_{CP-BIS} ≤-2kg (n=154)		Pair T-test	0kg < DW_{CP-BIS} < 1kg (n=405)		2kg ≤ DW_{CP-BIS} (n=246)		Pair T-test
Feature	Mean	SD	Mean	SD	p-value	p-value	SD	Mean	SD	p-value
Age	63.76	12.66	66.34	13.17	0.211673	65.89	12.18	63.72	11.64	0.008188
Gender(%)	204(47%)	0.50	65(42%)	0.50	0.37281	157(39%)	0.49	80(33%)	0.47	0.015267
Height (cm)	159.88	8.40	160.96	8.25	0.263094	161.94	8.03	163.88	9.29	0.004188
Weight (kg)	60.78	11.47	64.68	13.08	0.007522	59.61	11.23	59.07	10.68	0.497721
Vintage of HD (day)	745.55	753.38	854.78	850.92	0.653565	874.61	848.06	833.20	893.90	0.052677
DM	239(55%)	0.50	95(62%)	0.49	0.819401	247(61%)	0.49	161(65%)	0.48	0.341385
HTN(%)	351(81%)	0.39	111(72%)	0.45	0.003609	343(85%)	0.36	181(74%)	0.44	5.66E-05
Hb (g/dL)	10.33	1.44	10.29	1.63	0.790732	10.21	1.39	9.54	1.64	7.61E-06
Total Protein (g/dL)	6.41	0.66	6.35	0.72	0.940049	6.35	0.66	6.11	0.86	6.97E-05
Albumin (g/dL)	3.51	0.56	3.26	0.68	0.054126	3.44	0.54	3.13	0.63	4.07E-09
BUN (mg/dL)	57.68	22.61	56.10	25.18	0.131003	57.86	23.17	52.02	23.79	0.007318
Cr (mg/dL)	8.07	3.35	7.40	4.08	0.135468	8.08	3.17	6.76	3.26	5.01E-05
Tca (mg/dL)	8.36	0.76	8.40	0.76	0.308357	8.38	0.74	8.36	0.95	0.757956
P (mg/dL)	4.49	1.49	4.32	1.63	0.064399	4.38	1.61	4.03	1.47	0.042321
Na (mEq/L)	137.19	3.92	137.19	4.12	0.689801	137.10	4.04	136.89	4.13	0.882701
K (mEq/L)	4.73	0.87	4.69	0.97	0.720405	4.82	0.89	4.57	0.96	0.004298
Cl (mEq/L)	101.66	5.29	101.73	5.14	0.607159	101.27	5.08	100.78	5.00	0.444655
TBW (L)	31.51	7.13	32.72	7.93	0.133793	31.64	6.81	33.00	7.67	0.000451
ECW (L)	14.99	3.48	15.16	3.69	0.919144	15.24	3.20	17.14	3.92	1.98E-12
ICW (L)	16.47	4.10	17.57	5.15	0.018393	16.38	4.05	15.88	4.30	0.76851
E/I	0.93	0.16	0.90	0.22	0.056883	0.95	0.16	1.11	0.21	1.16E-19
BMI (kg/m^2)	23.73	3.75	24.87	4.45	0.039558	22.62	3.85	21.99	3.28	0.399772
LTI (kg/m^2)	13.22	3.22	13.79	4.34	0.081457	12.94	3.31	12.38	3.51	0.338029
FTI (kg/m^2)	9.76	4.75	10.74	5.09	0.257509	8.74	4.49	7.73	4.23	0.014575
LTM (kg/m^2)	34.31	10.53	35.93	12.74	0.109772	34.35	10.39	33.55	11.22	0.843426
FAT (kg)	19.53	9.64	22.25	10.69	0.123561	17.80	9.33	16.51	9.59	0.138475
ATM (kg)	22.86	10.53	25.65	12.47	0.040081	21.47	10.94	19.04	10.22	0.031125

BCM (kg)	18.99	7.04	20.06	8.72	0.119062	18.89	7.08	18.10	7.58	0.77238
DWBIS (kg)	59.29	11.04	64.37	12.97	0.000264	57.62	10.83	54.69	10.52	0.048247
$OH(L)_{BIS}$ (kg)	1.50	1.93	0.63	2.78	0.000307	1.89	1.78	4.38	2.70	1.42E-27
DWCP (kg)	58.90	11.04	60.13	13.15	0.372368	58.04	10.84	58.85	10.17	0.055239
DWCP-BIS (kg)	-0.40	0.25	-4.24	2.39	1.19E-44	0.41	0.26	4.16	2.30	9.89E-71
OH(L)CP (kg)	1.88	1.97	4.55	3.51	1.69E-12	1.58	2.31	0.22	3.18	9.72E-07

Abbreviations: SD, standard deviation; HD, Hemodialysis; DM, diabetes mellitus; HTN, hypertension; Hb, hemoglobin; BUN, blood urea nitrogen; Cr, serum creatinine; TCa, serum total calcium; P, serum phosphorus; Na, sodium; K, serum potassium; Cl, serum chloride; TBW, total body water; ECW, extracellular water; ICW, intracellular water; E/I, extracellular water to intracellular water ratio; BMI, body mass index; LTI, lean tissue index; FTI, fat tissue index; lean tissue mass, LTM; FAT, fat mass; ATM, adipose tissue mass; BCM, body cell mass; DW, dry weight; OH, overhydration.

A

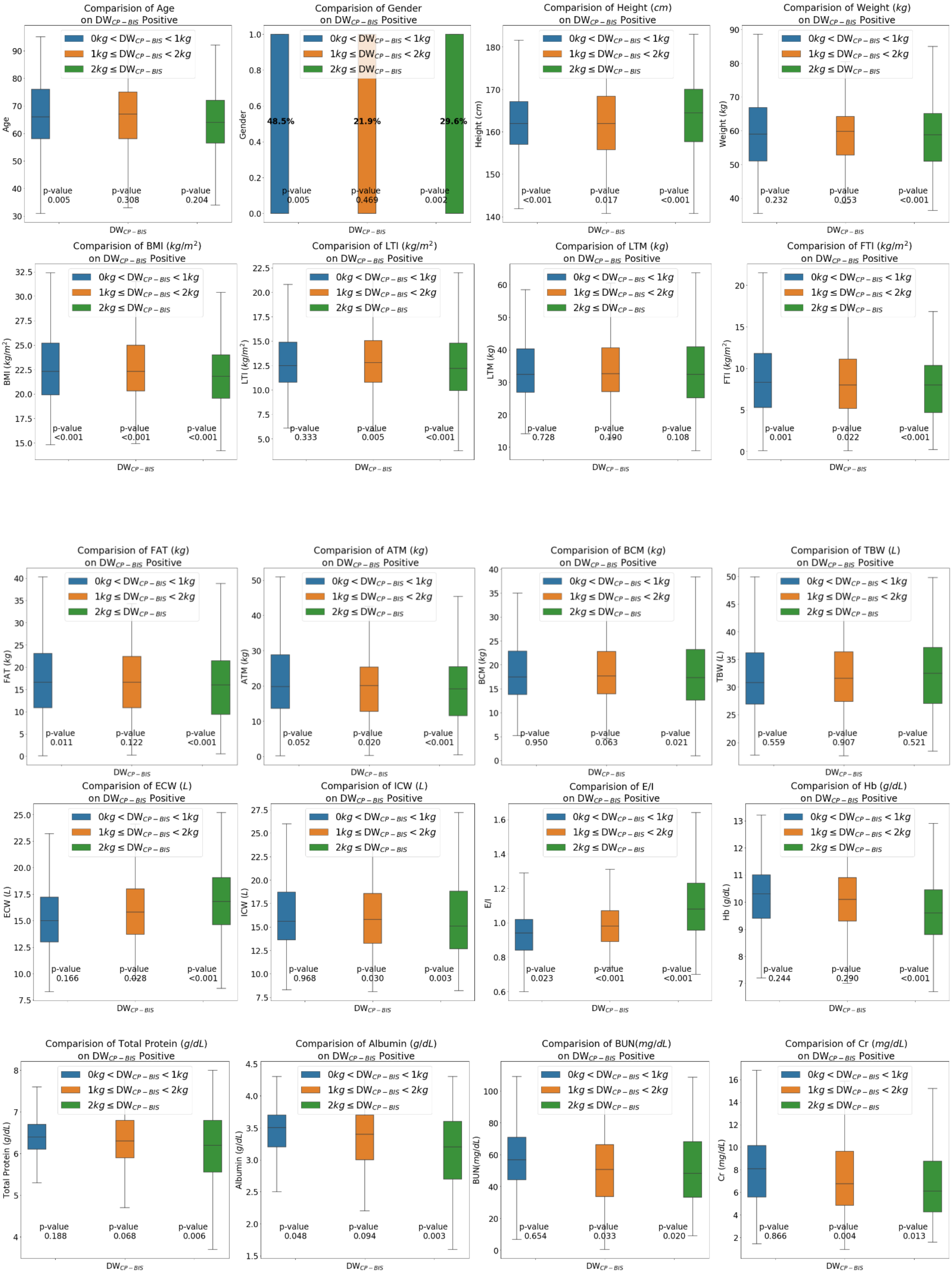
Comparision of Age on DW_{CP-BIS} Positive
$0kg < DW_{CP-BIS} < 1kg$
$1kg \leq DW_{CP-BIS} < 2kg$
$2kg \leq DW_{CP-BIS}$
Age
p-value 0.005
p-value 0.308
p-value 0.204
DW_{CP-BIS}
Comparision of Gender on DW_{CP-BIS} Positive
Gender
48.5%
21.9%
29.6%
p-value 0.005
p-value 0.469
p-value 0.002
Comparision of Height (cm) on DW_{CP-BIS} Positive
Height (cm)
p-value <0.001
p-value 0.017
p-value <0.001
Comparision of Weight (kg) on DW_{CP-BIS} Positive
Weight (kg)
p-value 0.232
p-value 0.053
p-value <0.001
Comparision of BMI (kg/m^2) on DW_{CP-BIS} Positive
BMI (kg/m^2)
p-value <0.001
p-value <0.001
p-value <0.001
Comparision of LTI (kg/m^2) on DW_{CP-BIS} Positive
LTI (kg/m^2)
p-value 0.333
p-value 0.005
p-value <0.001
Comparision of LTM (kg) on DW_{CP-BIS} Positive
LTM (kg)
p-value 0.728
p-value 0.190
p-value 0.108
Comparision of FTI (kg/m^2) on DW_{CP-BIS} Positive
FTI (kg/m^2)
p-value 0.001
p-value 0.022
p-value <0.001
Comparision of FAT (kg) on DW_{CP-BIS} Positive
FAT (kg)
p-value 0.011
p-value 0.122
p-value <0.001
Comparision of ATM (kg) on DW_{CP-BIS} Positive
ATM (kg)
p-value 0.052
p-value 0.020
p-value <0.001
Comparision of BCM (kg) on DW_{CP-BIS} Positive
BCM (kg)
p-value 0.950
p-value 0.063
p-value 0.021
Comparision of TBW (L) on DW_{CP-BIS} Positive
TBW (L)
p-value 0.559
p-value 0.907
p-value 0.521
Comparision of ECW (L) on DW_{CP-BIS} Positive
ECW (L)
p-value 0.166
p-value 0.028
p-value <0.001
Comparision of ICW (L) on DW_{CP-BIS} Positive
ICW (L)
p-value 0.968
p-value 0.030
p-value 0.003
Comparision of E/I on DW_{CP-BIS} Positive
E/I
p-value 0.023
p-value <0.001
p-value <0.001
Comparision of Hb (g/dL) on DW_{CP-BIS} Positive
Hb (g/dL)
p-value 0.244
p-value 0.290
p-value <0.001
Comparision of Total Protein (g/dL) on DW_{CP-BIS} Positive
Total Protein (g/dL)
p-value 0.188
p-value 0.068
p-value 0.006
Comparision of Albumin (g/dL) on DW_{CP-BIS} Positive
Albumin (g/dL)
p-value 0.048
p-value 0.094
p-value 0.003
Comparision of BUN(mg/dL) on DW_{CP-BIS} Positive
BUN(mg/dL)
p-value 0.654
p-value 0.033
p-value 0.020
Comparision of Cr (mg/dL) on DW_{CP-BIS} Positive
Cr (mg/dL)
p-value 0.866
p-value 0.004
p-value 0.013

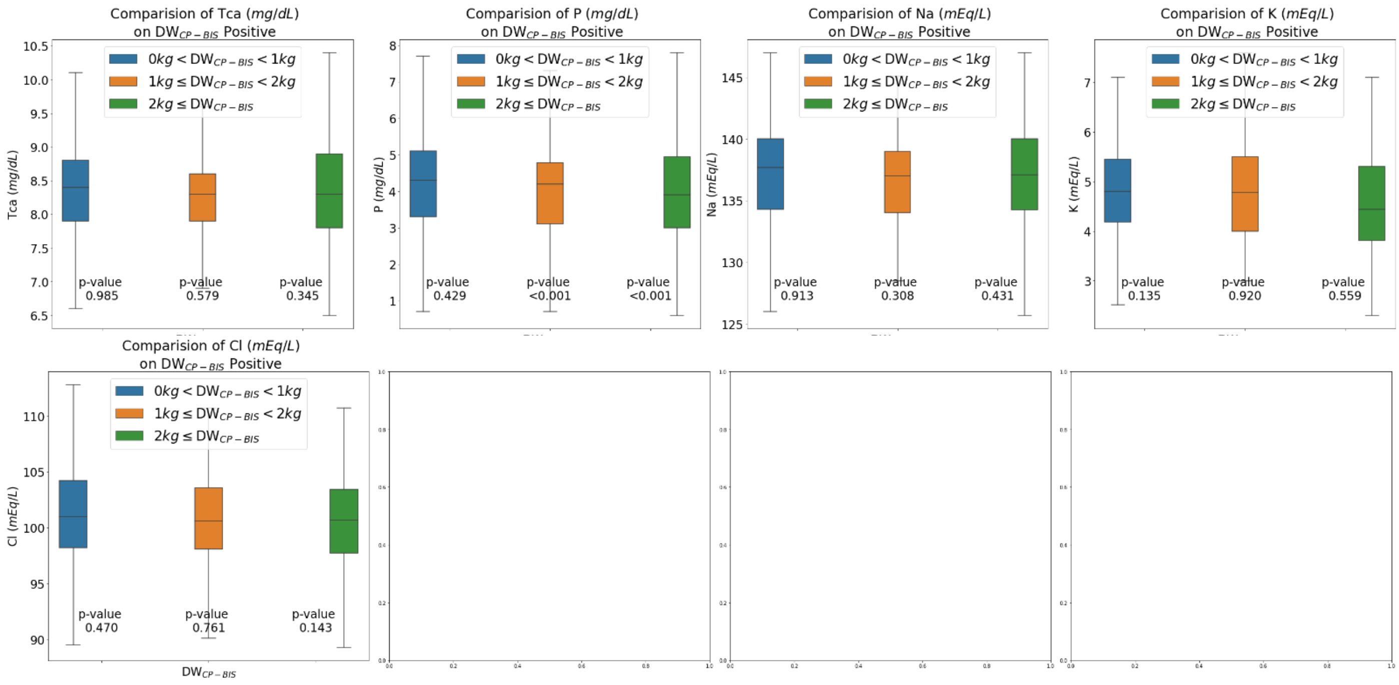
Comparision of Tca (mg/dL) on DW_CP−BIS Positive
Comparision of P (mg/dL) on DW_CP−BIS Positive
Comparision of Na (mEq/L) on DW_CP−BIS Positive
Comparision of K (mEq/L) on DW_CP−BIS Positive
Comparision of Cl (mEq/L) on DW_CP−BIS Positive
0kg < DW_CP−BIS < 1kg
1kg ≤ DW_CP−BIS < 2kg
2kg ≤ DW_CP−BIS
Tca (mg/dL)
p-value 0.985
p-value 0.579
p-value 0.345
P (mg/dL)
p-value 0.429
p-value <0.001
p-value <0.001
Na (mEq/L)
p-value 0.913
p-value 0.308
p-value 0.431
K (mEq/L)
p-value 0.135
p-value 0.920
p-value 0.559
Cl (mEq/L)
p-value 0.470
p-value 0.761
p-value 0.143
DW_CP−BIS

B

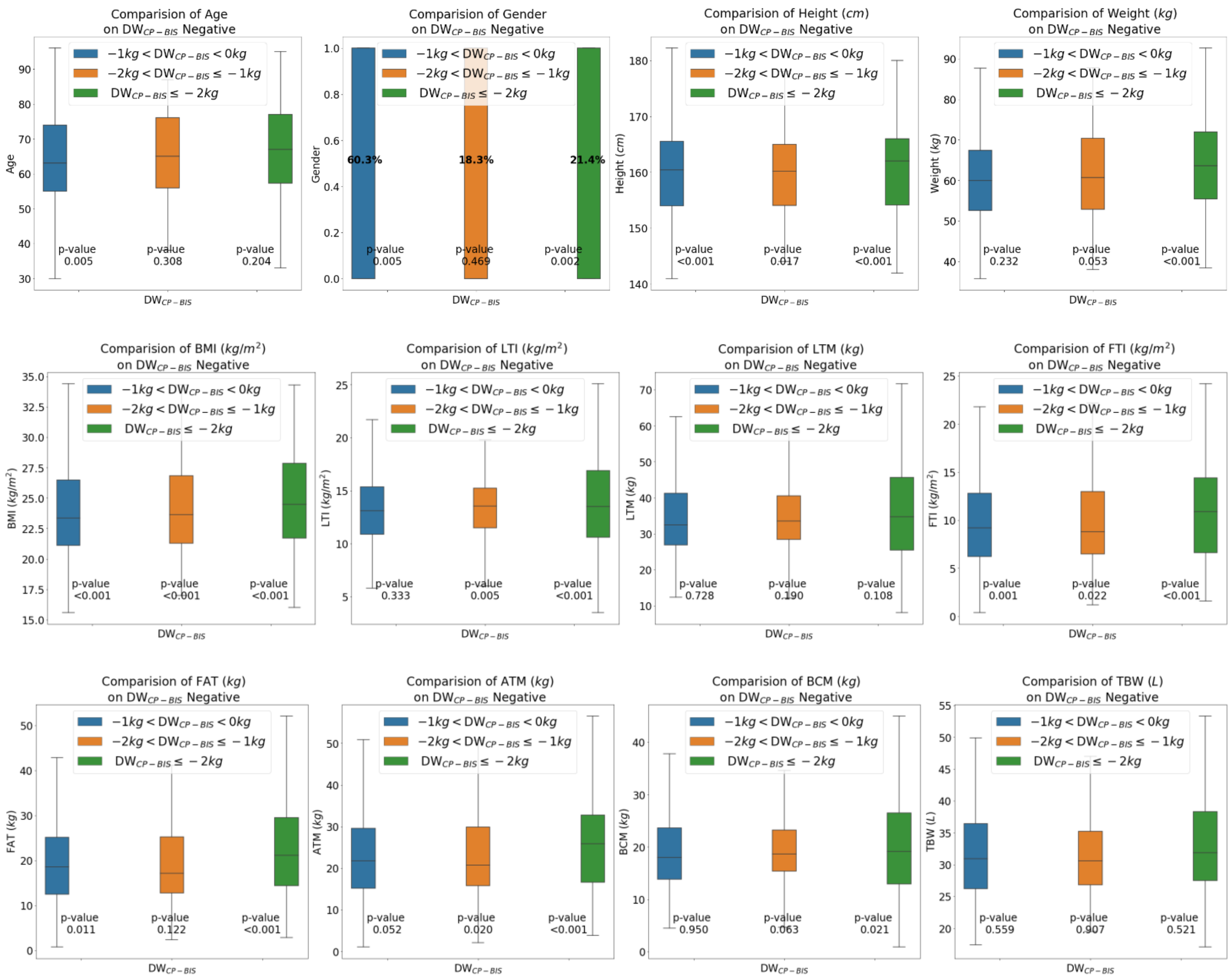
Comparision of Age on DW_CP−BIS Negative
Comparision of Gender on DW_CP−BIS Negative
Comparision of Height (cm) on DW_CP−BIS Negative
Comparision of Weight (kg) on DW_CP−BIS Negative
−1kg < DW_CP−BIS < 0kg
−2kg < DW_CP−BIS ≤ −1kg
DW_CP−BIS ≤ −2kg
Age
p-value 0.005
p-value 0.308
p-value 0.204
Gender
60.3%
18.3%
21.4%
p-value 0.005
p-value 0.469
p-value 0.002
Height (cm)
p-value <0.001
p-value 0.017
p-value <0.001
Weight (kg)
p-value 0.232
p-value 0.053
p-value <0.001
Comparision of BMI (kg/m²) on DW_CP−BIS Negative
Comparision of LTI (kg/m²) on DW_CP−BIS Negative
Comparision of LTM (kg) on DW_CP−BIS Negative
Comparision of FTI (kg/m²) on DW_CP−BIS Negative
BMI (kg/m²)
p-value <0.001
p-value <0.001
p-value <0.001
LTI (kg/m²)
p-value 0.333
p-value 0.005
p-value <0.001
LTM (kg)
p-value 0.728
p-value 0.190
p-value 0.108
FTI (kg/m²)
p-value 0.001
p-value 0.022
p-value <0.001
Comparision of FAT (kg) on DW_CP−BIS Negative
Comparision of ATM (kg) on DW_CP−BIS Negative
Comparision of BCM (kg) on DW_CP−BIS Negative
Comparision of TBW (L) on DW_CP−BIS Negative
FAT (kg)
p-value 0.011
p-value 0.122
p-value <0.001
ATM (kg)
p-value 0.052
p-value 0.020
p-value <0.001
BCM (kg)
p-value 0.950
p-value 0.063
p-value 0.021
TBW (L)
p-value 0.559
p-value 0.907
p-value 0.521
DW_CP−BIS

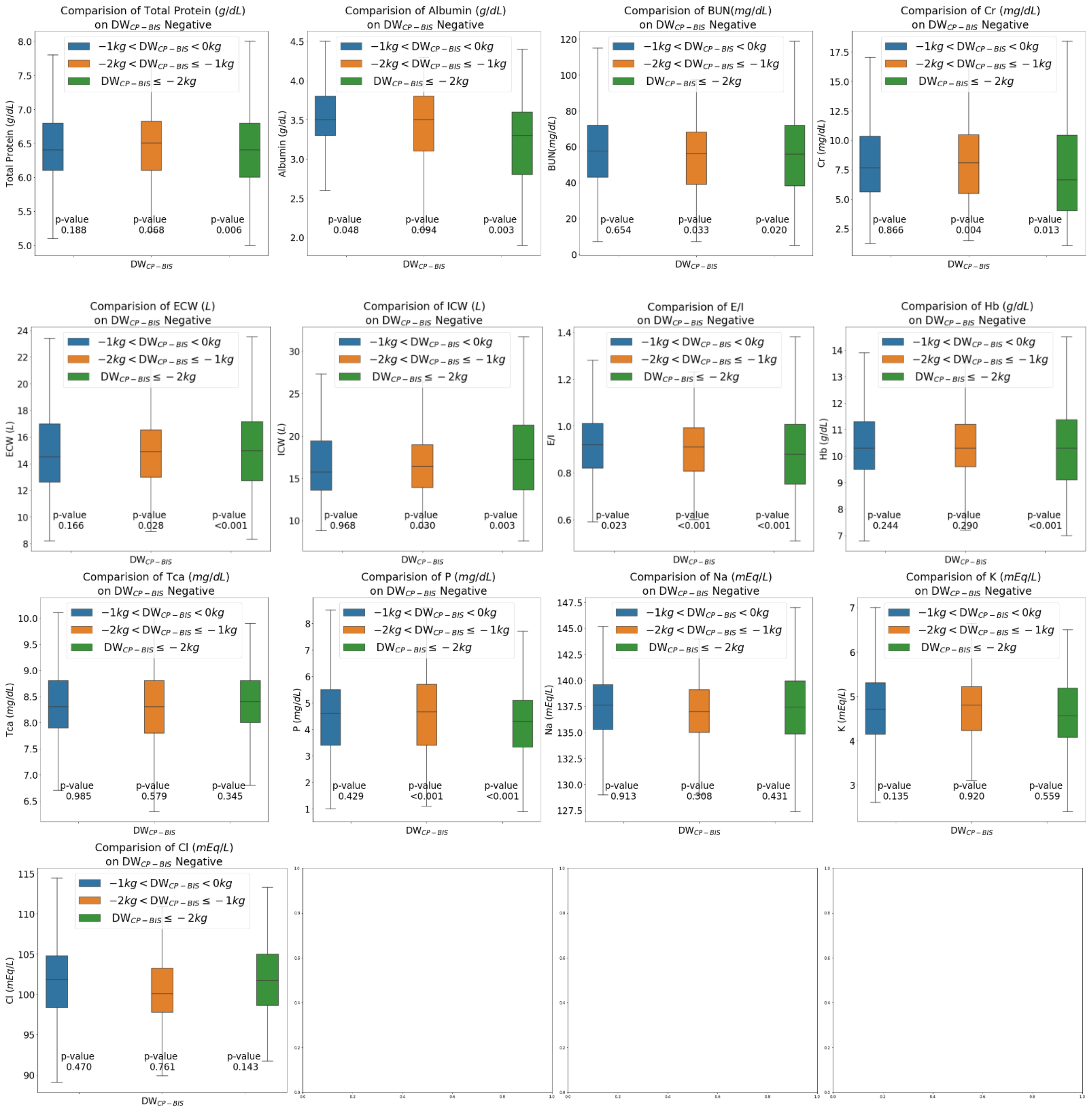

Figure S1. (A) Clinical Parameters as degrees of differences in Positive DW_{CP-BIS} groups. (B) Clinical Parameters as degrees of differences in Negative DW_{CP-BIS} groups.

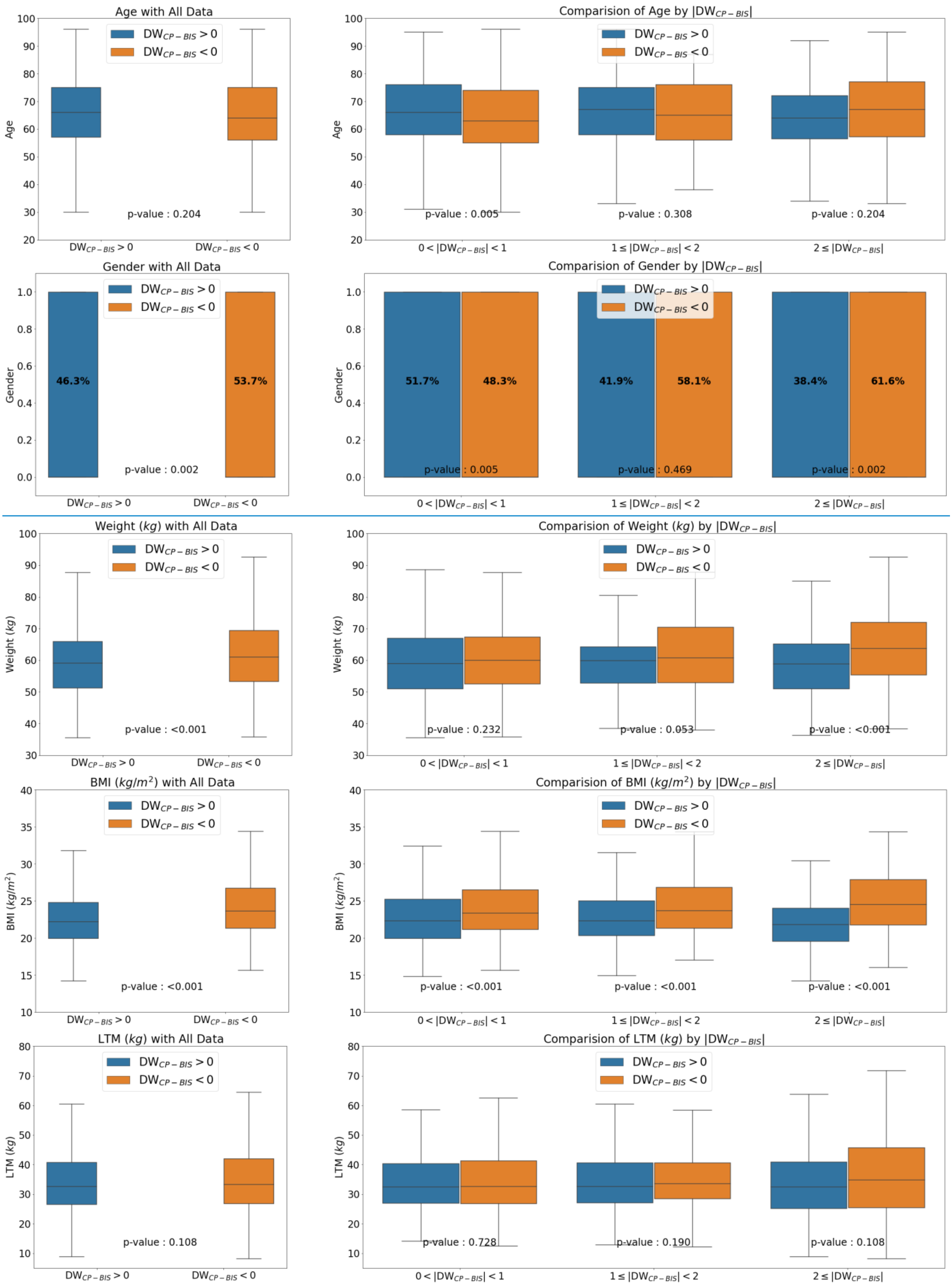

Age with All Data
Comparision of Age by $|DW_{CP-BIS}|$
$DW_{CP-BIS} > 0$
$DW_{CP-BIS} < 0$
Age
p-value : 0.204
p-value : 0.005
p-value : 0.308
p-value : 0.204
$0 < |DW_{CP-BIS}| < 1$
$1 \leq |DW_{CP-BIS}| < 2$
$2 \leq |DW_{CP-BIS}|$
Gender with All Data
Comparision of Gender by $|DW_{CP-BIS}|$
Gender
46.3%
53.7%
p-value : 0.002
51.7%
48.3%
41.9%
58.1%
38.4%
61.6%
p-value : 0.005
p-value : 0.469
p-value : 0.002
Weight (kg) with All Data
Comparision of Weight (kg) by $|DW_{CP-BIS}|$
Weight (kg)
p-value : <0.001
p-value : 0.232
p-value : 0.053
p-value : <0.001
BMI (kg/m^2) with All Data
Comparision of BMI (kg/m^2) by $|DW_{CP-BIS}|$
BMI (kg/m^2)
p-value : <0.001
p-value : <0.001
p-value : <0.001
p-value : <0.001
LTM (kg) with All Data
Comparision of LTM (kg) by $|DW_{CP-BIS}|$
LTM (kg)
p-value : 0.108
p-value : 0.728
p-value : 0.190
p-value : 0.108

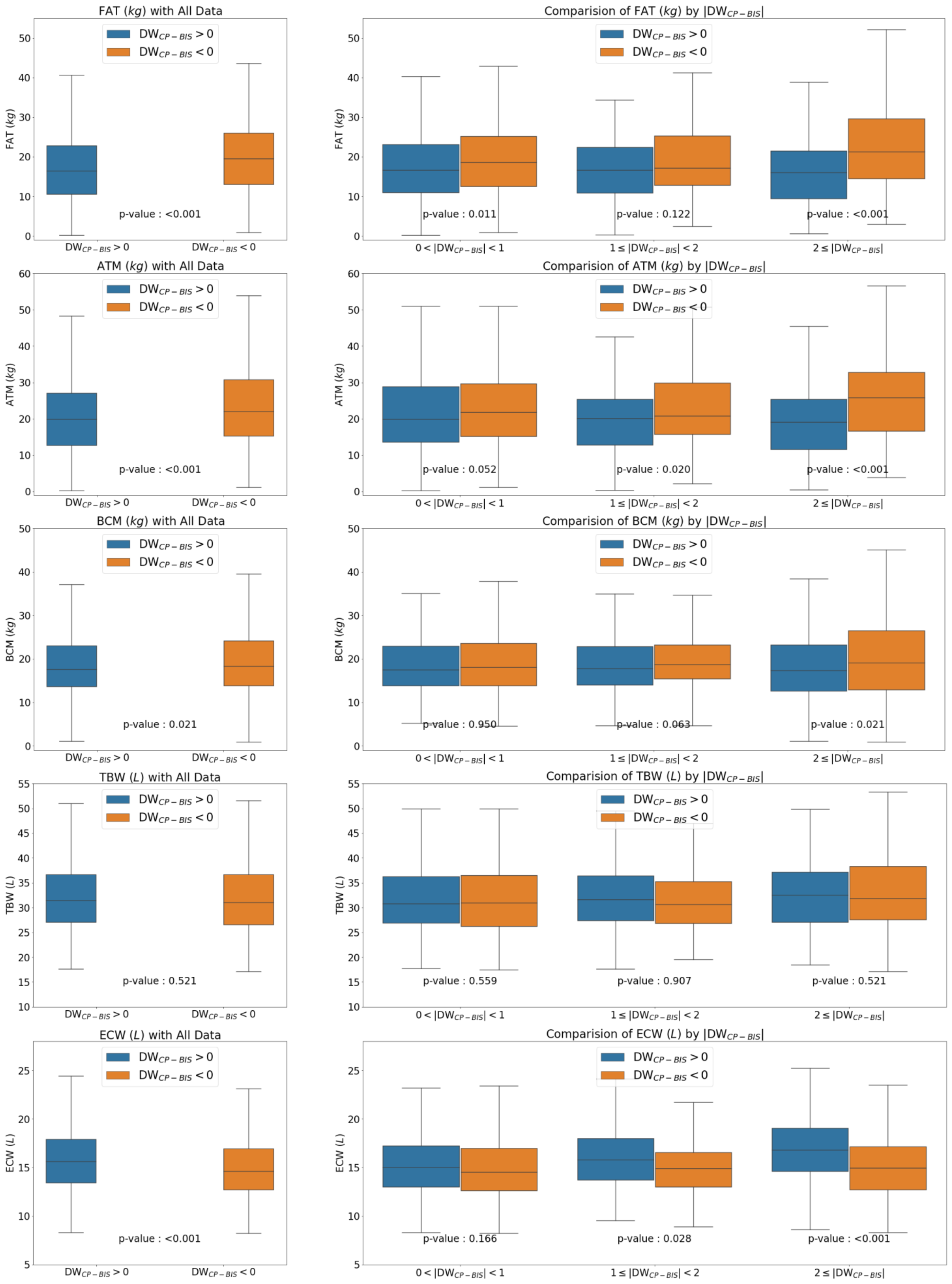
FAT (kg) with All Data
DW_CP−BIS > 0
DW_CP−BIS < 0
FAT (kg)
p-value : <0.001
Comparision of FAT (kg) by |DW_CP−BIS|
p-value : 0.011
p-value : 0.122
p-value : <0.001
0 < |DW_CP−BIS| < 1
1 ≤ |DW_CP−BIS| < 2
2 ≤ |DW_CP−BIS|
ATM (kg) with All Data
ATM (kg)
p-value : <0.001
Comparision of ATM (kg) by |DW_CP−BIS|
p-value : 0.052
p-value : 0.020
p-value : <0.001
BCM (kg) with All Data
BCM (kg)
p-value : 0.021
Comparision of BCM (kg) by |DW_CP−BIS|
p-value : 0.950
p-value : 0.063
p-value : 0.021
TBW (L) with All Data
TBW (L)
p-value : 0.521
Comparision of TBW (L) by |DW_CP−BIS|
p-value : 0.559
p-value : 0.907
p-value : 0.521
ECW (L) with All Data
ECW (L)
p-value : <0.001
Comparision of ECW (L) by |DW_CP−BIS|
p-value : 0.166
p-value : 0.028
p-value : <0.001

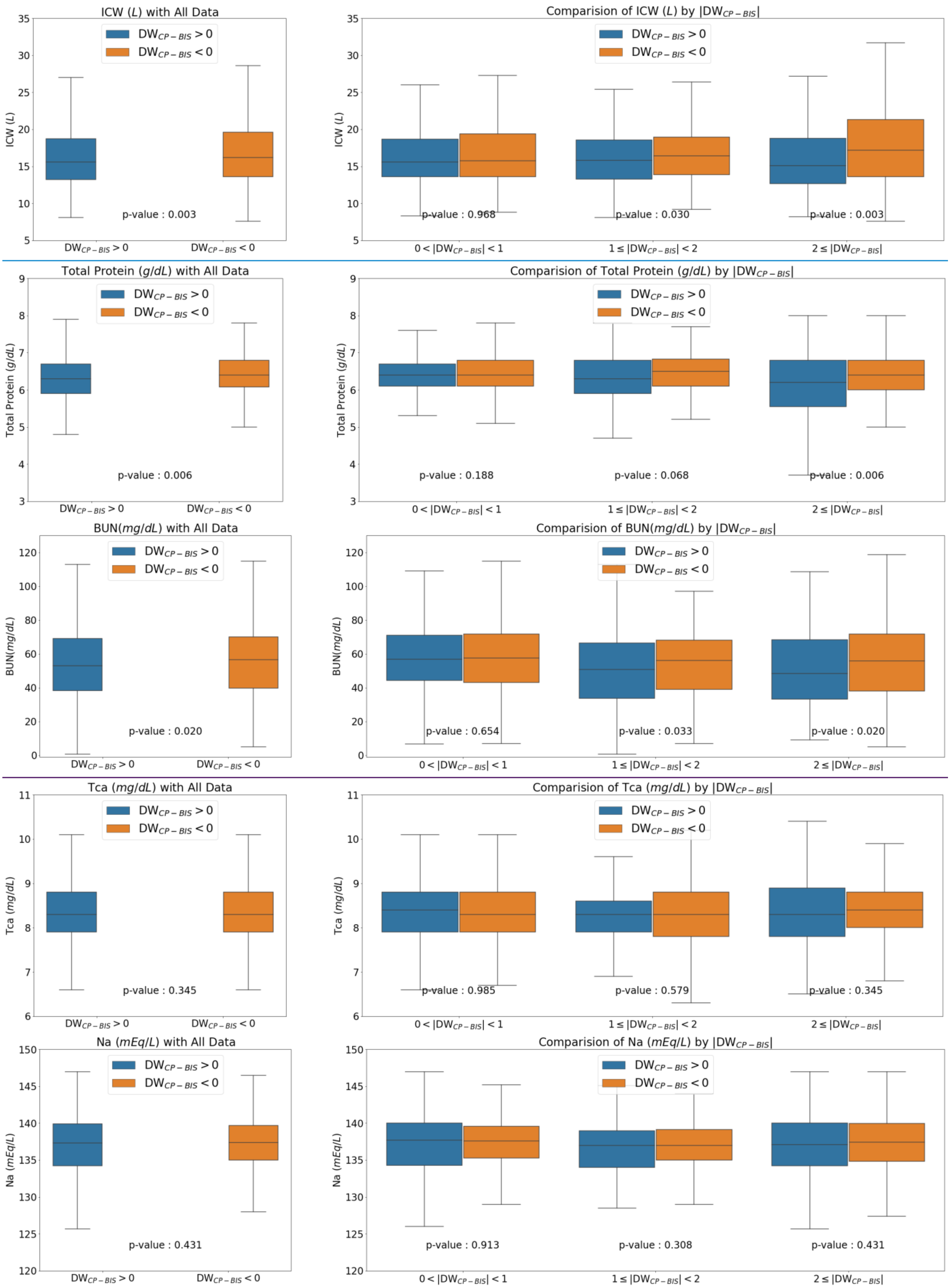

ICW (L) with All Data
Comparision of ICW (L) by |DW_CP−BIS|
DW_CP−BIS > 0
DW_CP−BIS < 0
ICW (L)
p-value : 0.003
p-value : 0.968
p-value : 0.030
p-value : 0.003
0 < |DW_CP−BIS| < 1
1 ≤ |DW_CP−BIS| < 2
2 ≤ |DW_CP−BIS|
Total Protein (g/dL) with All Data
Comparision of Total Protein (g/dL) by |DW_CP−BIS|
Total Protein (g/dL)
p-value : 0.006
p-value : 0.188
p-value : 0.068
p-value : 0.006
BUN(mg/dL) with All Data
Comparision of BUN(mg/dL) by |DW_CP−BIS|
BUN(mg/dL)
p-value : 0.020
p-value : 0.654
p-value : 0.033
p-value : 0.020
Tca (mg/dL) with All Data
Comparision of Tca (mg/dL) by |DW_CP−BIS|
Tca (mg/dL)
p-value : 0.345
p-value : 0.985
p-value : 0.579
p-value : 0.345
Na (mEq/L) with All Data
Comparision of Na (mEq/L) by |DW_CP−BIS|
Na (mEq/L)
p-value : 0.431
p-value : 0.913
p-value : 0.308
p-value : 0.431

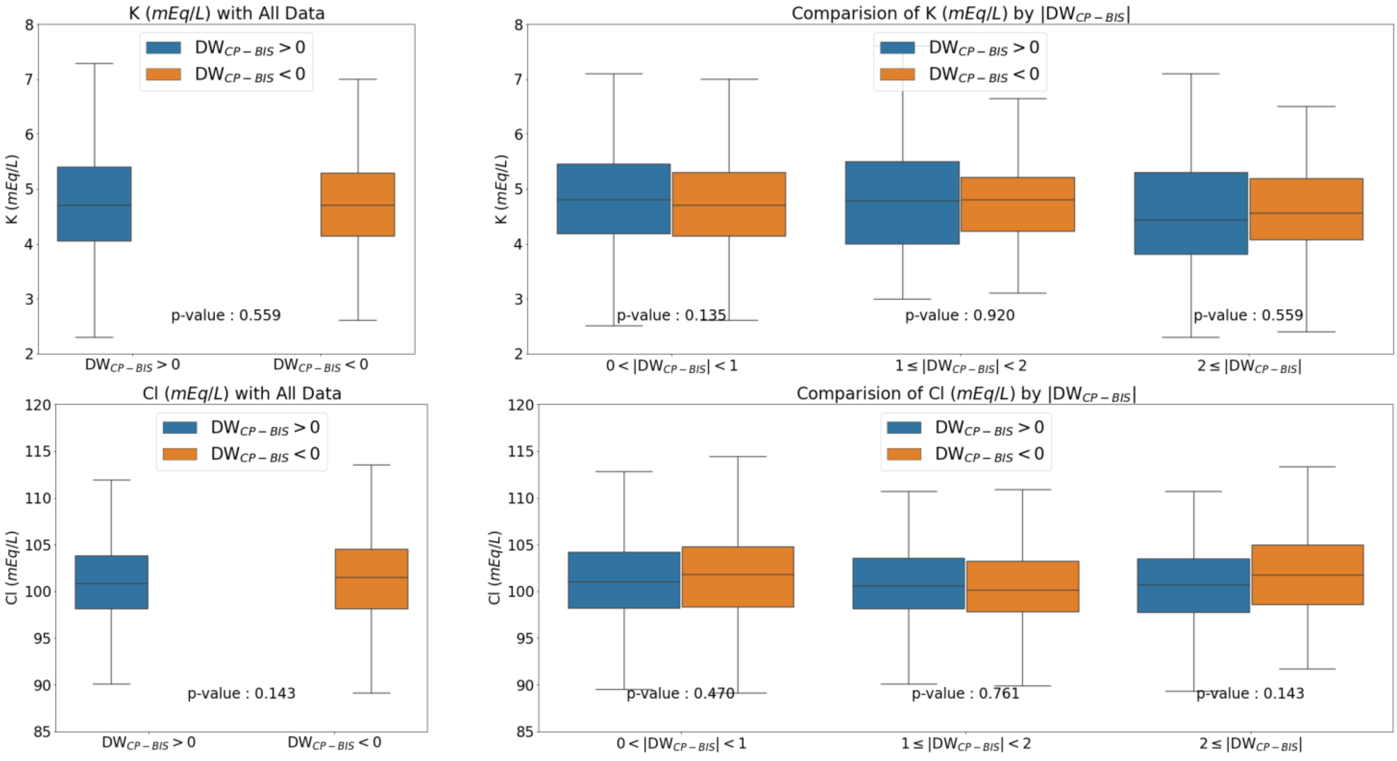

Figure S2. Comparison of clinical parameter by differences of $DW_{CP\text{-}BIS}$.
